# Calling RNF168 to action

**DOI:** 10.15698/cst2018.05.135

**Published:** 2018-05-10

**Authors:** Somaira Nowsheen, Zhenkun Lou

**Affiliations:** 1 Mayo Clinic Medical Scientist Training Program, Mayo Medical School and Mayo Graduate School, Mayo Clinic, Rochester, MN 55905, USA.; 2 Division of Oncology, Mayo Clinic, Rochester, MN 55905, USA.

**Keywords:** DNA double strand break repair, DNA damage response, L3MBTL2, RNF168, RNF8, ubiquitin, E3 ligase, ATM, phosphorylation, MDC1

## Abstract

Genomic stress leads to various forms of DNA damage, of which DNA double strand breaks (DSBs) are the most lethal. An army of signaling molecules is called to action as soon as these DNA breaks are detected. Various protein modifications, such as phosphorylation and ubiquitination, are an integral part of the reaction. While phosphorylation activates various proteins, ubiquitin (Ub) adducts typically act as docking sites for DNA repair factors. The response to DNA DSB starts with the protein kinase ATM phosphorylating various substrates including MDC1 and histone H2AX. This mediator protein, MDC1, then recognizes phosphorylated histone H2AX and amplifies the damage response. The E3 ligase, RNF8, recognizes and binds to phosphorylated MDC1. RNF8 then modifies an unknown protein to call a second ubiquitin ligase, RNF168, into action. It has been recognized that these two ubiquitin ligases are recruited sequentially but there is an unknown linker protein between them. These two ubiquitin ligases are crucial to the formation of DSB-associated ubiquitin conjugates and, as a result, there has been long standing interest in the field in identifying the link between the two factors. In this paper we identify lethal(3) malignant brain tumor like 2 (L3MBTL2) as the substrate of RNF8 (Nowsheen S, et al. Nat Cell Biol 20:455-464, 2018). We report that ATM-mediated phosphorylation of the polycomb group like protein L3MBTL2 and subsequent interaction with MDC1 brings it to the vicinity of the DNA lesion. RNF8 acts upon this phosphorylated L3MBTL2 and generates K63-linked polyubiquitin chains. This modified substrate is subsequently recognized by RNF168 and tethers the protein to the DNA lesion. RNF168 then ubiquitinates proteins such as histone H2A and H2AX to further amplify the damage response and recruit repair proteins such as BRCA1 and 53BP1 (Figure 1).

 It is interesting to note that MDC1 acts as a node for the recruitment of both RNF8 and L3MBTL2 and brings these two factors into proximity. As we and others have shown previously, MDC1 is at the core of the DNA damage response, playing an early and important role in this signaling cascade. We show that the FHA domain of MDC1 is required for L3MBTL2 retention at DNA lesions. Ours is among a handful of reports showing that, in addition to phosphorylated threonine residues, the FHA domain of MDC1 recognizes phosphorylated serine residues. This suggests that there is promiscuity to the interactions of this domain of MDC1.

**Figure 1:**
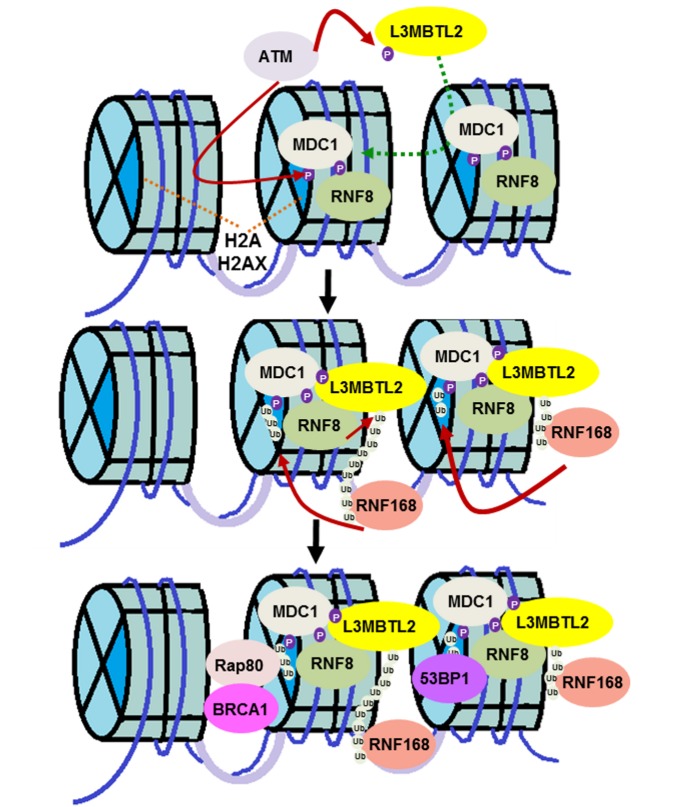
FIGURE 1: L3MBTL2 links RNF8 and RNF168 in the DNA double strand break response. The protein kinase ATM phosphorylates L3MBTL2, which recruits it to the DNA lesion by promoting the interaction between MDC1 and L3MBTL2. L3MBTL2 is subsequently ubiquitinated by RNF8, which acts as a docking site for RNF168, thereby recruiting the ubiquitin ligase to the damage site. RNF168, in turn, ubiquitinates H2A-type histones to amplify the DNA damage response and recruit downstream DNA repair proteins for proper DSB signaling.

It had been proposed that the linker histone H1 plays a role similar to L3MBTL2, linking the two ubiquitin ligases. However, in our hands, we were unable to see an appreciative change in RNF168 foci formation, a surrogate marker for its recruitment to DNA lesions, after histone H1 knockdown. Given how abundant histone H1 is, it is possible that this discrepancy is due to differences in knockdown efficiency. We and others have also been unable to detect poly-ubiquitination of histone H1 which is a requisite for it to be a binding platform for RNF168, given that RNF8 generates poly-ubiquitin chains. Thus, we believe that L3MBTL2 is the linker protein between RNF8 and RNF168.

A number of chromatin remodelers have been linked to DNA damage response. However, this is the first report linking L3MBTL2 to this complex pathway. Previously, L3MBTL2 has been shown to be in the atypical PRC complex. We show that the role of L3MBTL2 in DNA DSB repair is independent of the PRC complex. It remains to be seen what dictates the various roles of L3MBTL2. We also found that all four methyl binding (MBT) domains of L3MBTL2 are required for its function in DNA damage response. The MBT domain has previously been reported to be important for recognition of methylated lysine residues and transcriptional repression. It is unclear whether deletion of individual MBT domains disrupts L3MBTL2 structure, or L3MBTL2 interacts with methylated residues of a yet to be determined substrate that is important for its function. Interestingly, another member of the L3MBT family of proteins, L3MBTL1, has also been implicated in the regulation of 53BP1 accrual at DNA damage sites. L3MBTL1 specifically binds H4K20me2 and blocks 53BP1 recruitment. In response to DNA damage, L3MBTL1 is ubiquitinated and removed from chromatin to facilitate 53BP1 recruitment. Therefore, L3MBTL1 and L3MBTL2 have opposite roles in 53BP1 accrual to DNA damage sites. Since chromatin marks and conformation regulate many essential pathways such as DNA repair, transcription, DNA replication, and protein activity and accrual, it is not entirely surprising that a polycomb protein mediates this crucial link between RNF8 and RNF168.

Clearly, the intricacies of this chromatin network remain to be elucidated. An important question that remains is how cells decide which repair pathway to follow. A number of repair factors, such as CtIP, RIF1, and REV7, have been identified over the last few years that supposedly dictate the choice of DNA repair pathway. It also remains to be seen what determines the specificity of the ubiquitination binding through different Ub binding domains. In addition, how are Ub conjugates fine-tuned and are there other new modifications that are important for DSB repair? These are some of the remaining unanswered questions that linger in the field.

In summary, our results indicate that RNF8-mediated K63-linked poly-ubiquitination of L3MBTL2 at DNA damage sites acts as a loading platform for the recruitment of RNF168, thereby, enabling ubiquitination of H2A and subsequent recruitment of repair factors.

